# Potential safety signals for antibacterial agents from the Brazilian national pharmacovigilance database (Vigimed/VigiFlow)

**DOI:** 10.3389/fphar.2022.948339

**Published:** 2022-09-20

**Authors:** Luiza Hoehl Loureiro Alves Barbosa, Alice Ramos Oliveira Silva, Ana Paula D’Alincourt Carvalho-Assef, Elisangela Costa Lima, Fabricio Alves Barbosa da Silva

**Affiliations:** ^1^ Institute of Biosciences, Federal University of the State of Rio de Janeiro, Rio de Janeiro, Brazil; ^2^ Observatório de Vigilância e Uso de Medicamentos, Faculty of Pharmacy, Federal University of Rio de Janeiro, Rio de Janeiro, Brazil; ^3^ Laboratório de Pesquisa em Infecção Hospitalar, Instituto Oswaldo Cruz, Fiocruz, Rio de Janeiro, Brazil; ^4^ Scientific Computing Program, Oswaldo Cruz Foundation, Rio de Janeiro, Brazil

**Keywords:** pharmacovigilance, adverse drug reaction reporting systems, anti-bacterial agents, drug-related side effects and adverse reactions, safety signals, spontaneous reports

## Abstract

Antibacterial drugs are a widely used drug class due to the frequency of infectious diseases globally. Risks knowledge should ground these medicines’ selection. Data mining in large databases is essential to identify early safety signals and to support pharmacovigilance systems. We conducted a cross-sectional study to assess adverse drug events related to antibiotics reporting between December 2018 and December 2021 in the Brazilian database (Vigimed/VigiFlow). We used the Reporting Odds Ratio (ROR) disproportionality analysis method to identify disproportionate reporting signals (SDR), referring to statistical combinations between drugs and adverse events. Vancomycin was the most reported antibiotic (*n* = 1,733), followed by ceftriaxone (*n* = 1,277) and piperacillin and tazobactam (*n* = 1,024). We detected 294 safety signals related to antibacterials. We identified azithromycin leading in the number of safety signals (*n* = 49), followed by polymyxin B (*n* = 25). Of these, 95 were not provided for in the drug label and had little or no reports in the medical literature. Three serious events are associated with ceftazidime and avibactam, a new drug in the Brazilian market. We also found suicide attempts as a sign associated with amoxicillin/clavulanate. Gait disturbance, a worrying event, especially in the elderly, was associated with azithromycin. Our findings may help guide further pharmacoepidemiologic studies and monitoring safety signals in pharmacovigilance.

## Introduction

Individual case safety reports (ICSRs) from the spontaneous reporting system provide an essential information source for studying adverse drug events (ADEs) (Li et al., 2022). The database screening may be used to discover unknown drug-event pairs associated with signals of disproportionate reporting (Dijkstra et al., 2020). The evaluation of a signal encompasses quantitative and qualitative aspects (Meyboom et al., 1997; Hauben and Aronson, 2009). Meyboom and collaborators define a signal is more than just a statistical association, it consists of a hypothesis based on data and arguments (Meyboom et al., 1997) According to the World Health Organization (WHO), a safety signal is an information on a new or known ADE that may be caused by a medicine and is typically generated from more than a single report of a suspected event. A signal does not imply a direct causal relationship between an event and a drug but added with more information (consistent data, biological plausibility, association strength, for example) raises a hypothesis that requires further assessment (World Health Organization, 2022a). Regarding the quantitative aspect, statistical techniques (cluster analysis, link analysis, deviation detection, and disproportionality assessment) can be used to determine and assess the strength of ADE signals by the disproportional analysis (Montastruc et al., 2011).

Several measures of disproportionality reporting are available, and some studies propose direct comparisons of methods using synthetic, noise-free data (Zorych et al., 2013; Dijkstra et al., 2020). The reporting odds ratio (ROR) is the disproportionality method used by Eudravigilance e UMC Uppsala (World Health Organization, 2022a; European Medicines Agency, 2022) and performs well in most cases.

Low and middle-income countries (LMIC) face challenges in establishing efficient pharmacovigilance systems capable of generating data to inform health policies and practices (Kiguba et al., 2021). In Brazil, the largest country in South America, a new ADE notification system was deployed in 2018 (Vogler et al., 2020) by the National Health Surveillance Agency (ANVISA). Vigimed was the name given in Brazil to Vigiflow, a web-based ICSR management system developed by UMC, which replaced Notivisa due to system instability, an inability to import data series or export the database for analysis, and a lack of analytical and statistical tools (Vogler et al., 2020).

Data mining in pharmacovigilance databases may guide 1) additional analytical studies in specific populations that may be more susceptible to ADE occurrence and 2) epidemiologic studies for risk quantification and risk-minimization activities (Emanuel Raschi, 2016). Previous studies from our group found relevant signals of disproportionate reporting in pediatric and oncologic care from the former Brazilian database (Notivisa) (Barcelos et al., 2019; Vieira et al., 2020).

Antibacterials are a strategic drug class for pharmacoepidemiologic studies due to the frequency and their use consequences. These medicines have been associated with ADEs, microbial resistance, morbidity, and mortality worldwide (Tamma et al., 2017; Montastruc et al., 2021; Li et al., 2022). In addition, cultural factors can influence antibiotic use (Touboul-Lundgren et al., 2015). Brazil is one of the countries with the highest consumption of these drugs (World Health Organization, 2018) which raised on COVID-19 disease pandemic (Silva et al., 2021; Ul Mustafa et al., 2021).

We aimed to identify and analyze potential safety signals related to antibacterial agents for systemic use from the Brazilian electronic system for the spontaneous report (Vigimed/VigiFlow) from 2018 to 2021. This study is the first to analyze signals of disproportionate reporting involving antibacterials based on data obtained from the new Brazilian electronic reporting system.

## Materials and methods

We conducted a registry-based cross-sectional study (European Medicines Agency, 2021). We collected data from the ADE spontaneous reports Brazilian database (Vigimed/Vigiflow) between January and March 2022. The Vigimed platform is currently available in the ANVISA database (Agência Nacional de Vigilância Sanitária, 2022c). We included all reports of antibacterials for systemic according to Anatomical Therapeutic Chemical Classification System - ATC J01) related to 1 December 2018, and 31 December 2021 period (World Health Organization, 2020).

Antibacterials were classified by the fifth level of the Anatomical Therapeutic Chemical (ATC) Code (World Health Organization, 2020). We also considered AWaRe Classification to compare ADE reports by antibiotics with different levels (three groups) of microbial resistance (Habarugira et al., 2021). The Access group includes antibiotics with a lower potential for resistance than the other groups. They are first or second-choice empirical treatments for infectious syndromes. Watch group antibiotics should be prioritized as critical targets of administration and monitoring programs. The Watch group has 11 antibiotics in the WHO Model List of Essential Medicines. Antibiotics in the Reserve group should be treated as a “last resort”. These drugs are vital points of antimicrobial stewardship programs. Seven antibiotics from the reserve group make up the WHO Model List of Essential Medicines (World Health Organization, 2019, 2021).

We compared case/non-case where cases were ICSR with ADE. We created a database containing 1) drug names, 2) reported ADE, and 3) the number of notifications of each ADE for each target drug. According to the European Medicines Agency, we did not consider drug-event pairs with less than three notifications and notifications dealing with medication errors (Aronson, 2009; European Medicines Agency, 2018b).

Concerning statistical analyses, we used the Reporting Odds Ratio (ROR) disproportionality analysis method, as implemented by the EudraVigilance Data Analysis System (EVDAS) used by the European Medicines Agency (European Medicines Agency, 2018a). We used the ROR measure to identify disproportionate reporting signals (SDR), referring to statistical combinations between drugs and ADEs. This method assumes that when a signal (involving a specific ADE) is associated with a drug, it indicates that the ADEs reported more frequently in association with this drug than other drugs (European Medicines Agency, 2006). The ROR calculation considers its 95% confidence interval and the number of individual cases (Van Puijenbroek et al., 2002).

The ROR measure is defined by the formula [(a.d)/(c.b)], where:

• “a” indicates the number of reports that list the target drug P and the target ADE R;
• “b” indicates the number of reports that list the target drug P but not the target ADE R;
• “c” indicates the number of reports that list the target ADE R but not the drug P;
• Finally, “d” indicates the number of reports that do not list the target ADE R or drug P.

When the inferior limit of the ROR’s 95% confidence interval is greater than 1, the association between ADE and the target drug is considered statistically significant (Rothman et al., 2004). Therefore, we consider it an SDR. A situation occurs when c = 0 or all database reports containing a target ADE are associated with only one drug. In this case, there is a division by zero, and it is not possible to calculate the ROR. In this situation, the ROR value is arbitrarily set at 99.9 to reflect the presence of a possible SDR. We also differentiate in the following analysis the cases where 3 ≤ *a* <5 and a ≥5.

We assess previous information about safety signals found in the drug labels (summary of product characteristics - SPC) available on the ANVISA website (Agência Nacional de Vigilância Sanitária, 2022b). All safety signals not mentioned in SPCs were tabulated. Additionally, we investigated the existence of any complementary data that could reinforce the suspicion of a causal relationship between the antibiotic and the observed event in studies published in the following databases: UpToDate, Pubmed, Embase, Web of Science, and Medline. The search was performed using the drug name according to ATC and Preferred Terms (PT) (International Council for Harmonisation of Technical Requirements for Pharmaceuticals for Human Use, 2020).

## Results

**FIGURE 1:**
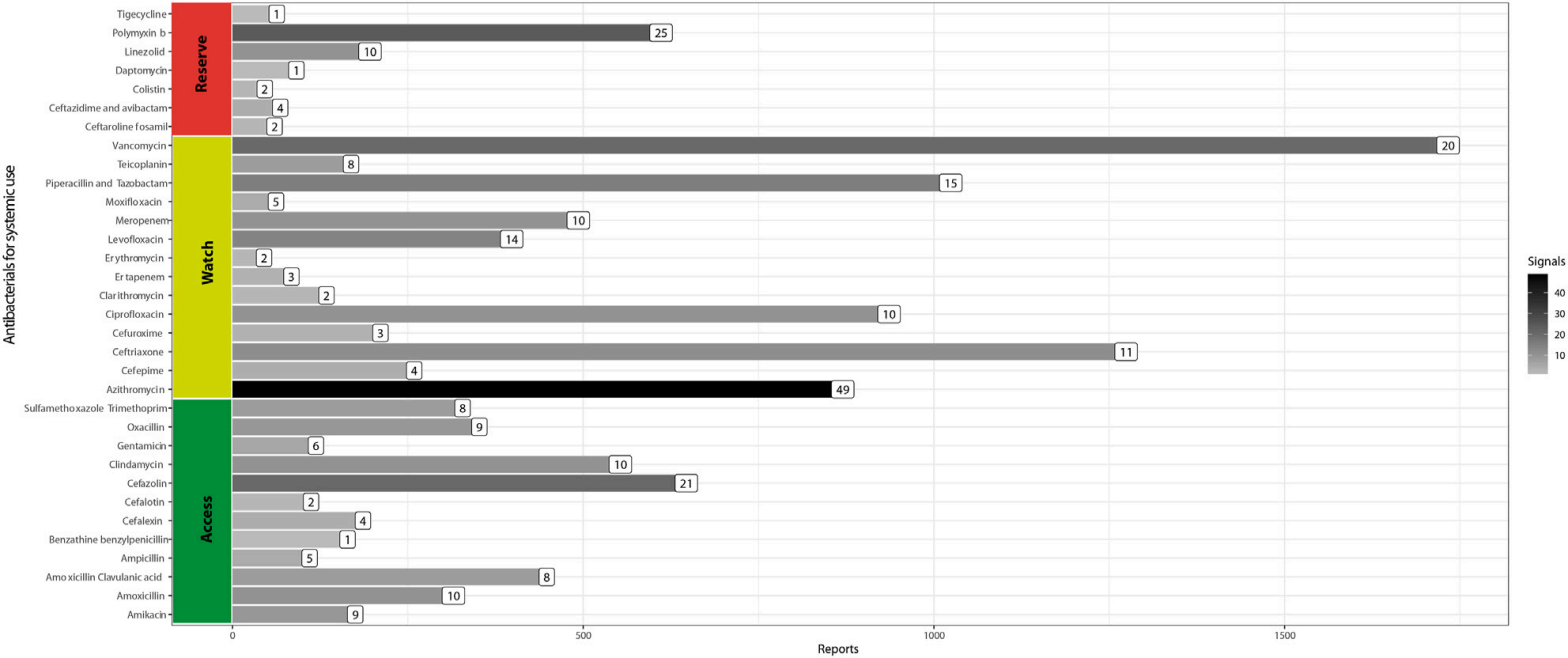
Adverse drug event reports and safety signals involving systemic antibacterials (Brazil, 2018–2021).

We found 12,665 ADE reports involving 53 antibacterials for systemic use (Group J01) between 1 December 2018, and 31 December 2021, in the Vigimed/VigiFlow database (Brazil). The most reported antibiotics were: vancomycin (*n* = 1,733), ceftriaxone (*n* = 1,274), piperacillin/tazobactam (*n* = 1,024), ciprofloxacin (*n* = 936), and azithromycin (*n* = 870) (Figure 1).

We obtained 294 safety signals. Azithromycin (*n* = 49) and polymyxin B (*n* = 25) were the main antibiotics involved in these signals, as shown by the bar color density in Figure 1. For 67.7% of pairs, the event reported was already described in the label of the respective antibiotic. Table 1 presents the 95 signals, few or not reported in the literature.

**TABLE 1:** Signals not reported on drug labels or post marketing (Brazil, 2022).

ATC Code	Drug	ADE	ROR	SDR Intensity	Previous description
Reserve					
J01DD52	Ceftazidime/avibactam	Death	144.77	a ≥3 a <5	not described
		Depressed Level of Consciousness	23.12		not described
		Sepsis	392.15		not described
J01DF01	Aztreonam	Tremor		a>=3 e c = 0	not described
J01XB02	Polymyxin b	Depressed Level of Consciousness	6.62	a ≥5	not described
		Dyspnoea	2.69		not described
		Acute Respiratory Failure	16.53		not described
		Oxygen Saturation Decreased	2.51		not described
		Cardio-Respiratory Arrest	4.94	a ≥3 a <5	not described
J01DI02	Ceftaroline fosamil	Flushing	6.47	a ≥3 a <5	not described
Watch					
J01FA10	Azithromycin	Oropharyngeal pain	23.81	a ≥5	not described
		Decreased Immune Responsiveness	5.08		not described
		Covid-19	16.18		not described
		Asthmatic Crisis	19.5		not described
		Immunodeficiency	81.58		not described
		Peripheral Swelling	81.58		not described
		Influenza	17.53		not described
		Drug Ineffective	2.41		Literature^1^
		Gait Disturbance	10.19		not described
		Pneumonia	7.32		not described
		Dysaesthesia	20.39		not described
		Asthma	18.08	a ≥3 a <5	not described
		Depression	18.08		not described
		Dysphonia	9.04		not described
		Dyspepsia	4.51		not described
		Pharyngitis	20.32		not described
		Depressed Mood	27.12		not described
		Urinary Tract Infection	3.87		not described
		Osteoarthritis	40.65		not described
		Weight Decreased	4.51		not described
		Blood Pressure Increased	8.12		not described
		Condition Aggravated	4.06		not described
		Rhinitis	8.13		not described
		Rhinorrhoea	40.65		not described
		Sinusitis	10.16		not described
		Productive Cough	20.32		not described
		Psoriatic Arthropathy	54.26		not described
Watch					
J01DD04	Ceftriaxone	Dyspnoea	2.03	a ≥5	not described
		Phlebitis	2.67		Literature^a^
		Papule	3.82		not described
		Cough	2.6		not described
J01FA09	Clarithromycin	Phlebitis	5.98	a ≥5	Literature^a^
J01DC02	Cefuroxime	Drug Ineffective	9.02	a ≥5	Literature^b^
J01MA02	Ciprofloxacin	Oedema Peripheral	3.58		not described
		Swelling	2.26	a ≥3 a <5	not described
		Vomiting	2.63	a ≥5	Literature^c^
J01MA12	Levofloxacin	Anxiety	4.87		Literature^d^
		Miliaria	6.19		not described
		Loss of Consciousness	46.4	a ≥3 a <5	not described
		Skin Lesion	8.62	a ≥5	Literature^d^
J01CR05	Piperacillin and Tazobactam	Angioedema	2.5		not described
		Decreased Appetite	5.73	a ≥5	not described
J01XA02	Teicoplanin	Skin Lesion	11.3		not described
		Lymphopenia	226.36	a ≥3 a <5	not described
J01GB01	Tobramycin	Eyelid Oedema	40.65	a ≥3 a <5	not described
J01MA14	Moxifloxacin	Petechiae	10.05	a ≥3 a <5	not described
Access					
J01GB06	Amikacin	Colitis	4.41	a ≥3 a <5	not described
J01CA04	Amoxicillin	Influenza	13.17	a ≥3 a <5	not described
		Drug Ineffective	3.21		Literature^e^
		Weight Increased	19.71		not described
		Pneumonia	6.95		not described
		Pyrexia	2.55		Literature^c^
		Suicide Attempt	49.59	a ≥5	not described
J01CR02	Amoxicillin Clavulanic acid	Asthenia	3.42	a ≥3 a <5	not described
		Chest Pain	5.12		not described
		Oedema Peripheral	5.45		not described
		Drug Ineffective	4.89	a ≥5	Literature^a^
		Pneumonia	11.78		not described
J01DB04	Cefazolin	Angioedema	3.42	a ≥5	not described
		Bronchospasm	5.22		not described
		Oedema	3.42		not described
		Eyelid Oedema	4.29		not described
		Lip Swelling	4.2		not described
		Laryngeal Oedema	4.27		not described
		Corneal Oedema	5.07		not described
		Hyperhidrosis	3.13		not described
		Papule	6.27		not described
Access					
J01DB04	Cefazolin	Skin Plaque	8.69	a ≥3 a <5	not described
		Tachycardia	2.81		not described
		Sneezing	15.04		not described
		Ocular Pyperaemia	4.69		not described
		Throat Irritation	7.51		not described
J01DB03	Cefalotin	Paraesthesia	4.07	a ≥3 a <5	not described
J01FF01	Clindamycin	Throat Tightness	13.18	a ≥3 a <5	Literature^d^
		Petechiae	3.04	a ≥5	not described
J01GB03	Gentamicin	Petechiae	3.74	a ≥3 a <5	not described
J01CF04	Oxacillin	Eosinophilia	3.69	a ≥5	not described
		Dermatitis	1.99		not described
		Chemical Phlebitis	7.1		Literature^a^
		Pyrexia	2.68		Literature^b^ ^,^ ^e^
		Pruritus	1.65		Literature^a^
		Hyperthermia	15.05	a ≥3 a <5	Literature^b^ ^,^ ^e^
		Hypoaesthesia	11.07		not described
		Urticaria Papular	11.7		not described
J01EE01	Sulfamethoxazole Trimethoprim	Urinary Tract Infection	7.6	a ≥3 a <5	not described

ADE, Adverse drug event; ROR, reporting odd ratio; SDR, signal of disproportionate reporting.

a Observational cohort study.

b Retrospective countrywide.

c Narrative review.

d Case report.

e Pharmacovigilance study.

Concerning serious safety signals, we found eight involving antibiotics classified as Reserve. Polymyxin B had five serious ADE (depressed level of consciousness, dyspnea, acute respiratory failure, oxygen saturation decreased, and cardio-respiratory arrest) not previously described. Death, depression level of consciousness, and sepsis with ceftazidime/avibactam, an antibiotic recently introduced in the Brazilian market, were also identified in our study (Table 1).

In watch antibiotics, gait disturbance, a worrying event, especially in the elderly, was associated with azithromycin. Suicide attempts with amoxicillin also is a serious and unprecedented potential safety signal. Ineffectiveness, described in other countries, was obtained for azithromycin, cefuroxime, amoxicillin, and amoxicillin/clavulanate (Table 1).

In the access class, we highlight hyperthermia as an event previously described for oxacillin (Table 1).

## Discussion

This first study involving the new Brazilian electronic notification system (Vigimed/VigiFlow) found 294 safety signals associated with disproportionate reporting for antibacterials of the three AWaRe classes. Some signals.

Ceftazidime/avibactam is a new antibiotic that began to be marketed in Brazil in 2018 and that seemed to be well tolerated (Cheng et al., 2020). This drug is restricted to infections with no other therapeutic option and is expected to have a lower frequency of use than other agents (Watch and Access) (World Health Organization, 2019). A study that analyzed adverse events from two phases II and III clinical trials did not report any serious adverse events (Cheng et al., 2020). In the report issued by the EMA in 2021, ceftazidime/avibactam was not related to any safety signal (European Medicines Agency, 2018c). However, we found three potentially serious signals related to ceftazidime-avibactam (Table 1). Other pharmacovigilance databases also received reports of the same ADE for this same drug. Eudravigilance and Vigiaccess contain eight and 19 reports of sepsis associated with the same drug, respectively (European Medicines Agency, 2018c; World Health Organization, 2022b). Five reports for the depressed level of consciousness and 82 for death were described in VigiAccess (World Health Organization, 2022b). Ceftazidime/avibactam was the suspicion drug for 123 deaths in the FDA Adverse Event Reporting System (FAERS) (Food and Drug Administration, 2021). Nevertheless, data that support the causality for these ADEs are not yet available.

We found many signals of azithromycin associated with infections and immune system disorders. The possible causal relationship between azithromycin and the reported infections has biological plausibility. The pathophysiological mechanism for this adverse effect refers to local microbiota modification by the azithromycin, eliminating bacteria commensals and allowing the growth and proliferation of fungi and opportunistic microorganisms (Seelig, 1966). For example, there is a risk of candidiasis caused by azithromycin (Wilton et al., 2003). Similarly, microbiological imbalance generated by the previous use of antibiotics might cause broad immunity and metabolism changes, leading to the recurrence of infections, such as pneumonia (Francino, 2015). Withal gait disorders contribute to reduced mobility, fall risk, diminished quality of life, and serious injuries, including significant fractures and head trauma (Pirker and Katzenschlager, 2017). Eventually, drugs cause gait disturbance (Kanao-Kanda et al., 2016), but we did not find gait disturbances as an adverse event associated with azithromycin in the medical literature. Nevertheless, a study reported akathisia (ADE typically attributed to antipsychotics) after exposure to azithromycin (Riesselman and El-Mallakh, 2015). Accumulating azithromycin in brain tissue can prove unpredictable effects (Jaruratanasirikul et al., 1996), a possible mechanism by which azithromycin causes gait disturbance. The pandemic caused by COVID-19 increased azithromycin consumption in Brazil (Del Fiol et al., 2022). This scenario may have contributed to the emergence of safety signals not previously observed (Meyboom et al., 1997) (Figure 1).

Our study showed ineffectiveness signals for two antibacterials classified as Watch (cefuroxime and azithromycin) and amoxicillin, azithromycin, and amoxicillin/clavulanate. The preferred terms “ineffective drug,” “therapeutic product effects decreased,” and “pathogen resistance” are triggers for monitoring microbial resistance in pharmacovigilance databases (Habarugira et al., 2021). We expect the investigation of these specific signals in observational studies in the Brazilian population.

We found five previously unreported signals associated with polymyxin B. Depressed level of consciousness and cardiorespiratory arrest may be secondary to hyponatremia, an adverse event associated with polymyxin B previously (Rodriguez et al., 1970). Drug interactions of polymyxin B with other drugs might cause respiratory symptoms, such as neuromuscular blockers and aminoglycosides (Pohlmann, 1966). Furthermore, in 1964, a report on respiratory arrest associated with polymyxin B infusion was published (Small, 1964). To our knowledge, there have been no other similar reports.

We described suicide attempts and amoxicillin as a signal in our analysis. We found no previous report of amoxicillin-related suicidal behavior in the medical literature. However, in the FAERS database, suicide attempt events associated with amoxicillin also were reported (*n* = 73) (Food and Drug Administration, 2021). Suicidal behavior is heterogeneous and multifactorial, influenced by complex interactions between biological, psychological, and social factors. Some antibiotics cause suicidal ideation by daedal neuropharmacological interactions involving the GABAergic receptor (Samyde et al., 2016). So, the existence of causal relations cannot be ruled out. In the same way, we did not find reports of the pair chest pain or asthenia with amoxicillin and clavulanate, but for this second ADE, there are 1111 reports of suspicion with this drug in the VigiAccess (World Health Organization, 2022b). Despite being an antimicrobial that has been on the market for a long time and is widely used in outpatient settings, the safety signals should be monitored due to its seriousness and the demographic, genetic, and nutritional patterns in different populations (World Health Organization, 2012).

Fluoroquinolones are antibiotics with numerous adverse effects well established by regulatory agencies (Samyde et al., 2016; Agência Nacional de Vigilância Sanitária, 2019; Food and Drug Administration, 2019). Notwithstanding, some ADE might be underestimated. According to levofloxacin labels, the incidence of anxiety associated with the drug occurs in less than 1%, and there is no report of loss of consciousness as a primary event associated with levofloxacin. However, levofloxacin may trigger important neurologic events (Mazzei et al., 2012; Scavone et al., 2020), resulting in loss of consciousness.

We described several signals without any mention in literature for some antibiotics (ceftaroline fosamil, meropenem, ceftriaxone, piperacillin and tazobactam, teicoplanin, tobramycin, moxifloxacin, and cefazolin) in Table 1. Such signals have less relevance for monitoring given their lower severity and lack of information to strengthen the hypothesis of a causal relationship. Despite everything, knowledge about pharmacovigilance is dynamic, and new data may be added in the future.

ANVISA’s Pharmacovigilance Management - Gerência de Farmacovigilância (GFARM)- evaluates notifications received at Vigimed/Vigiflow and forwards them to the WHO Uppsala Center. If the Uppsala Center emits any signal, GFARM initiates an investigation process. If the investigation confirms the causality between the event and drug exposure, GFARM alerts health professionals and drug users and establishes complementary regulatory measures (e.g., drug label change) (Agência Nacional de Vigilância Sanitária, 2022a).

We performed an exploratory analysis of crude data from the Vigimed/Vigiflow database to detect safety signals of antibacterials in a marked strategic period by initial reports in this new Brazilian database and increase of this class of drugs consumption for this population (Sartelli et al., 2020). Disproportionality analysis in pharmacovigilance databases is an effective method for the early detection of these signals, especially for rare ADEs (European Medicines Agency, 2022). Nevertheless, this tool has inherent limitations in data mining studies from spontaneous reporting systems. Some ADE may be underreported, and the absence of a clinical history prevents the analysis of the causality of the reported ADE. We use the Reporting Odds Ratio measure, an example of a frequentist approach for Disproportionality Analysis in pharmacovigilance. It is generally accepted that neither bayesian nor frequentist methods are better than the other (Harpaz et al., 2012; Huang et al., 2014). Frequentist approaches are more computationally efficient than Bayesian measures but may generate more false positives. The Bayesian methods also incorporate information about disproportionality and sample size in a single dimension. Nevertheless, none of the approaches can effectively address reporting biases or confounding in spontaneous reporting systems (Harpaz et al., 2012).

Besides that, every disproportionality measure might require a new reinvestigation of data, including pharmacodynamic analysis of biological plausibility (Montastruc et al., 2011). All in all, signal management consists of several pharmacovigilance processes: 1) signal detection, 2) prioritization, 3) validation, 4) evaluation, and 5) outcome documentation (European Medicine Agency, 2018). Although this analysis may have shown serious undetectable signals in other countries, our findings are limited to Brazil.Boletim de Farmacovigilância No13, 2020.

## Conclusion

Antibiotics were widely used during the COVID-19 pandemic, resulting in a suitable scenario to observe unreported safety signals. Our study detected 95 new safety signals. Three serious signals are associated with ceftazidime/avibactam, a drug recently introduced in the Brazilian market. Suicidal behavior was related to the commonly prescribed antibiotic in community infections, amoxicillin. Gait disturbance, a worrying event, especially in the elderly, was associated with azithromycin. Given the seriousness of these potential signals, we suggested further pharmacoepidemiologic studies for more investigation and monitoring by the regulatory agencies.

## Data Availability

Publicly available datasets were analyzed in this study. This data can be found here: https://www.gov.br/anvisa/pt-br/acessoainformacao/dadosabertos/informacoes-analiticas/notificacoes-de-farmacovigilancia.
